# Obesity and Atrial Fibrillation: Weighing the Evidence

**DOI:** 10.3390/jcm15208101

**Published:** 2026-10-19

**Authors:** Francis J. Ha, Jit Yoei Tang, Shaun Khanna, Adam J. Brown, Emily Kotschet, Nitesh Nerlekar

**Affiliations:** Victorian Heart Institute and Victorian Heart Hospital, Monash Health, Monash University, 631 Blackburn Road, Clayton, VIC 3168, Australia

**Keywords:** atrial fibrillation, obesity, epicardial adipose tissue, GLP-1 receptor agonists, weight management, lifestyle risk factor modification

## Abstract

Obesity is a major, modifiable risk factor for atrial fibrillation (AF). Potential mechanisms include haemodynamic and metabolic changes, inflammation, changes in epicardial adipose tissue (EAT) composition and cardiovascular–kidney–metabolic (CKM) syndrome. EAT is increasingly recognised as a dynamic, pro-arrhythmic fat depot with a possible bidirectional relationship with AF, whereby AF itself may modulate EAT independent of weight change. CKM syndrome also underlies the complex interplay between obesity and AF. Despite guideline recommendations for at least 10% weight reduction in obese patients with AF, recent randomised trials focusing on weight reduction have largely failed to demonstrate clinically meaningful improvements in AF outcomes compared with catheter ablation or usual care. Incretin-based therapies including glucagon-like peptide-1 receptor agonists (GLP-1 RAs) are a promising therapy offering sustained weight loss alongside broader cardiometabolic benefits, although randomised trials with AF as a primary endpoint are lacking. Comprehensive multidisciplinary lifestyle risk factor modification (LRFM) clinics that address cardiometabolic risk factors, AF education and medication adherence have shown benefit beyond weight reduction alone, although their delivery has been heterogeneous. This narrative review examines the evolving relationship between obesity and AF including a multidimensional cardiometabolic lens, EAT characterisation, contemporary randomised trials of weight reduction, and the emerging role of incretin-based therapies and LRFM clinics.

## 1. Introduction

Atrial fibrillation (AF) is the most common sustained cardiac arrhythmia, and its prevalence continues to rise alongside the global obesity epidemic. Obesity is an independent risk factor for AF underpinned by a constellation of mechanisms including haemodynamic remodelling [1], obstructive sleep apnoea (OSA) [2] and cardiovascular–kidney–metabolic (CKM) syndrome [3]. In part, the relationship between obesity and AF is mediated by epicardial adipose tissue (EAT) given its direct anatomical proximity to atrial myocardium [4]. Emerging evidence suggests a potential bidirectional relationship in which AF itself promotes EAT accumulation [5].

Current guidelines recommend ≥10% weight reduction in obese patients with AF [6,7]. This is a Class I, Level B-R recommendation for patients with AF and BMI > 27 kg/m^2^ as per the 2023 ACC/AHA/ACCP/HRS AF guidelines [6] and a Class I, Level B recommendation as per the 2024 ESC AF guidelines [7]. It is important to note that these guidelines were based on one relatively small randomised trial [8] and several other influential observational studies of weight reduction [9,10,11], rather than randomised trials testing a 10% threshold. Yet more recent randomised trials have either achieved only modest weight reduction or have failed to translate weight loss into consistent improvements in AF-related outcomes. In particular, the LOSE-AF and SORT-AF trials found no difference in AF burden and symptom severity with weight reduction versus usual care and the PRAGUE-25 trial demonstrated superiority of catheter ablation over weight reduction in improving AF freedom [12,13,14]. The emergence of glucagon-like peptide-1 receptor agonists (GLP-1 RAs) offers a unique opportunity to achieve weight reduction alongside broader cardiometabolic benefits in patients with AF [15]. Moreover, there is an increasingly recognised role of comprehensive lifestyle risk factor modification (LRFM) clinics in AF care beyond focusing on weight management alone as supported in several randomised clinical trials (RCTs) [16,17].

This narrative review examines potential mechanisms underlying the relationship between obesity and AF, evaluates contemporary trials concerning weight reduction, and appraises the evolving role of incretin-based therapies and comprehensive LRFM in AF management. This narrative review expands on the relationship between AF and EAT from previous work [5]. Here, we discuss EAT more briefly and instead present it within a broader framework in the context of obesity.

## 2. Search Criteria and Strategies

The evidence cited was assembled from searches of Ovid MEDLINE with MeSH terms, supplemented by the authors’ subject matter expertise. Examples of included MeSH terms were “atrial fibrillation”, “obesity”, “overweight”, “epicardial adipose tissue”, and “glucagon-like peptides”. The search was done between 4 August 2026 and 15 September 2026. Evidence was chosen based on judged relevance to the topic and quality of evidence. Though no formal systematic review framework was implemented, conflicting findings were analysed for their methodology and considered in their appropriate clinical context(s) before the authors came to a conclusion about how best to interpret those findings.

## 3. Obesity-Associated Mechanisms Promoting Atrial Fibrillation

**Figure 1:**
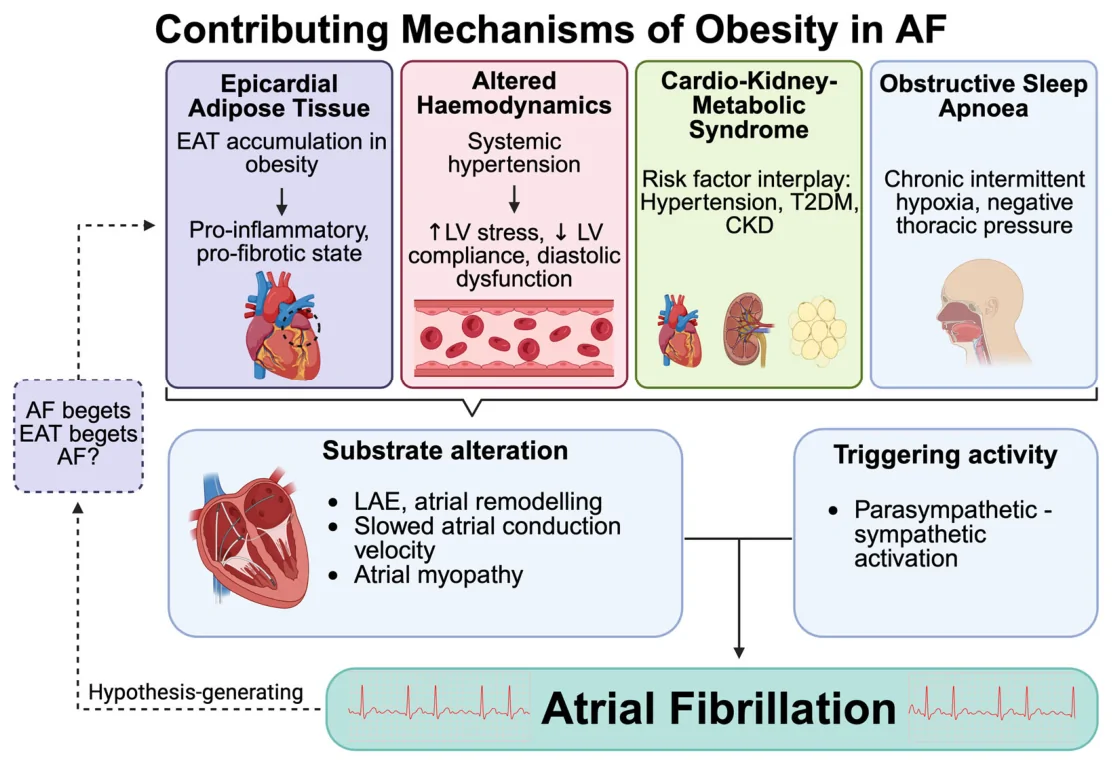
Potential mechanisms of obesity in AF. CKD, Chronic kidney disease. EAT, Epicardial adipose tissue. LV, Left ventricle. T2DM, Type 2 diabetes mellitus. Created using Biorender.com with licenced permission.

The relationship between obesity and AF is multifactorial (Figure 1). Underlying mechanisms include haemodynamic factors, dysfunctional metabolism, inflammation, fibrosis, cardiac ischaemia and OSA causing atrial remodelling as a substrate for AF [18,19,20]. Current guidelines support obesity as a risk factor [21] and provide a Class I recommendation for a weight-loss programme in overweight or obese patients [6,7].

Obesity is strongly associated with haemodynamic alterations such as hypertension, left ventricular (LV) wall stress, atrial remodelling and pulmonary arterial hypertension that increase the risk of AF [1]. These changes are also implicated in obesity-related cardiomyopathy [22]. A nationwide prospective cohort study in Korea found a dose-dependent relationship between the burden of hypertension and the risk of incident AF, with the highest burden of hypertension increasing the risk of incident AF by 27% [23]. Hypertension is associated with reduced LV compliance, diastolic dysfunction and LV hypertrophy, which results in atrial remodelling [19]. Blood pressure control is a Class I recommendation in AF management [6,7]. Left atrial enlargement is another link between obesity and AF [24,25]. A prospective cohort study from the Framingham cohort found the association between obesity and incident AF was mediated by left atrial enlargement [26]. A small prospective cohort study also found that indexed left atrial volume predicts AF recurrence post-ablation in obese patients [27]. Together, these suggest that obesity-related haemodynamics result in structural remodelling that, in part, underlies the pathogenesis of AF.

Metabolic dysfunction is also highly prevalent in obesity, which may promote AF through ectopic lipid deposition and lipotoxicity. When subcutaneous adipose storage capacity is exceeded, lipid “spillover” into EAT and the myocardium may promote oxidative stress, inflammation, fibrosis and abnormal calcium handling [28]. These changes contribute to atrial electrical and structural remodelling, providing both the substrate and triggers for AF.

Obesity is a significant risk factor for OSA and other sleep-disordered breathing, which is in turn a significant risk factor for AF [29,30,31]. Chronic intermittent hypoxia and negative thoracic pressure promote atrial stretch and remodelling [32]. Additionally, parasympathetic activation during apnoeic episodes in OSA followed by sympathetic activation due to hypoxia may precipitate AF itself [2,32]. This mechanism of parasympathetic–sympathetic activation is present in other forms of sleep-disordered breathing including central sleep apnoea [2]. Hence, screening for OSA is recommended in patients with resistant hypertension, pulmonary hypertension, recurrent AF and daytime sleepiness [33]. A retrospective cohort study found that OSA was associated with a two-fold increased risk of AF independent of obesity [34]. A meta-analysis of prospective cohort studies found that continuous positive airway pressure was associated with reduced AF recurrence (RR: 0.58, 95% CI: 0.51–0.67) [35]. However, certain randomised studies have failed to show benefits of OSA treatment. A randomised controlled trial examined patients with AF and Apnoea–Hypopnoea Index (AHI) > 5 who successfully underwent direct current cardioversion for AF and were randomised to receive either positive airway pressure (PAP) therapy or usual care [36]. The primary endpoint was time to AF recurrence in a 12-month follow-up period. The authors found no significant difference in time to AF recurrence (129.0 ± 166.5 days in the PAP group versus 109.3 ± 73.2 days in usual care, *p* = 0.98) between both treatment arms. Significant limitations were the small study size (*n* = 25) and the high drop-out rate (42% in the treatment arm and 31% in the control arm), which left 15 participants included in the analysis. This resulted in a large confidence interval and introduced the possibility of significant attrition bias. The subsequent atrial fibrillation, apnoea and airway study (A3 study) was a randomised controlled trial that consisted of two phases [37,38]. Phase 1 evaluated patients with paroxysmal AF and AHI ≥ 15 and randomised them to CPAP or usual care, with a mean BMI of 29.5 kg/m^2^ in the CPAP group and 29.4 kg/m^2^ in the control group [37]. The primary endpoint was change in AF burden measured on loop recorder as compared to baseline over a follow-up period of 5 months. The study found no significant between-group difference in time spent in AF (adjusted difference = −0.63 percentage points, 95% CI: −2.55 to 1.30, *p* = 0.52, *n* = 108). Phase 2 looked at patients from Phase 1 who underwent PVI and followed up with them for 12 months [38]. The primary endpoint was freedom from AF recurrence or atrial tachycardia on loop recorder after a 3-month blanking period. The study found no significant difference in the primary endpoint (OR: 1.0, 95% CI: 0.42–2.4, *p* = 1.00). Importantly, AHI in the CPAP group decreased from 26.7 ± 14.7 to 2.2 ± 1.7 before PVI and 1.7 ± 1.3 at follow-up post-PVI, indicating high adherence to therapy and its effectiveness in reducing OSA. Accordingly, OSA management remains a consideration in AF management and ESC guidelines list OSA management as a Class IIb recommendation [7].

Obesity is a common phenotype in CKM syndrome, which has received increasing recognition in the development of AF. The American Heart Association has formalised CKM syndrome which encompasses the cardiovascular system, chronic kidney disease (CKD) and metabolic syndrome [39]. Many obesity-linked risk factors for AF are also implicated as core components of CKM syndrome, such as hypertension, T2DM and CKD [39]. Scoring for CKM burden is usually determined by involvement of each domain or by CKM stage as defined by AHA guidelines [39]. Multiple cohort studies have demonstrated that CKM burden is associated with adverse outcomes, including all-cause mortality, MACEs, major adverse renal events (MAREs), major bleeding, thromboembolic events and heart failure hospitalisation in patients with AF [3,40,41]. In a large Taiwanese retrospective study, the authors found an inverse association between CKM burden and MAREs and all-cause mortality in patients with AF [42]. This was due to differences in BMI, where underweight patients (BMI < 18.5 kg/m^2^) had a worse prognosis compared to patients who had BMI ≥ 18.5 kg/m^2^. Notably, the kidney-only phenotype had the lowest BMI out of the other CKM phenotypes. This finding supports the obesity paradox, whereby in certain populations, having a lower BMI may be associated with reduced physiological reserve which may be associated with worse clinical outcomes [43,44,45]. Other explanations of the obesity paradox are confounding factors such as cigarette smoking (where smokers are likely to have a lower BMI while being at higher mortality risk) [46], reverse causality (where weight loss is due to illness and thus individuals with lower BMI are at greater mortality risk) [46] and sarcopenic obesity (where individuals may have apparently normal BMI but high fat percentage which increases mortality risk) [47,48]. This makes characterising the phenotype of obesity especially important for prognostication and management of AF. Indeed, another Taiwanese study found a stepwise increase in risk of adverse clinical outcomes with increasing CKM burden. This was likely due to the study including fewer underweight patients, with an average BMI of 23.70–28.15 kg/m^2^ [40]. Hence, clinical phenotyping of CKM syndrome needs to be undertaken to better estimate prognosis and guide management.

Lastly, obesity exhibits heterogeneous phenotypes between sexes that deserve further evaluation as to their impact on AF. Studies on AF (including the ones mentioned elsewhere in this review) have historically underrepresented women, which could limit the generalisability of their findings [49]. Obesity phenotypes differ between sexes, with men tending towards visceral adiposity and women towards subcutaneous adiposity [50,51,52]. These differences in phenotypes may be obscured by using BMI for the classification of obesity. Additionally, sex-specific differences in EAT exist. Women have been found to have lower total EAT volume and higher peri-atrial-EAT-to-total-EAT-volume ratio (P/T ratio) than men when matched for BMI in both overweight/obese and normal populations [53,54]. OSA may also manifest differently in women, which may cause it to be underdiagnosed [55]. The post-menopausal state of oestrogen deficiency may also contribute to increased susceptibility to cardiovascular diseases [56]. Overall, the role of obesity according to sex is still being understood in relation to incident AF, including the appropriate parameter for measurement of adiposity [57,58,59,60].

## 4. Evolving Perspective on Epicardial Adipose Tissue and AF

Epicardial adipose tissue is closely linked to AF. EAT predicts incident AF and post-operative AF beyond traditional measures of obesity such as body mass index (BMI) and waist circumference. In a large meta-analysis of observational studies, the association between BMI, waist circumference or waist/hip ratio and AF was a relative risk of 1.11–1.32 [61]. In contrast, EAT volume was associated with a 2.6× odds of AF and up to 5.4× odds of persistent AF. EAT is a dynamic fat depot capable of shifting between both a protective thermogenic state to a pro-inflammatory and pro-fibrotic state [62,63]. As sections of EAT lie adjacent to atrial myocardium, it communicates directly with the atria. Consequently, peri-atrial EAT correlates with slowed local atrial conduction velocity, fractionation of intra-cardiac electrograms and redistribution of gap junction protein Connexin 40 at cardiomyocyte borders [4].

Most research to date has examined EAT as a substrate and predictor of AF. But emerging clinical evidence points to a bidirectional relationship, in which AF itself may also promote EAT accumulation [5]. Three small-to-modest studies (382 total patients, 15 to 247 patients per study) examined EAT before and after catheter ablation. A small single-centre study retrospectively evaluated 15 patients who underwent thermal catheter ablation for AF where EAT was evaluated before and after ablation using magnetic resonance imaging (MRI) [64]. Over a median interval of 9.9 months between scans, left atrial (LA) EAT volume fell markedly from a median of 35.2 mL to 16.2 mL—a 54% relative reduction—despite only a minimal change in BMI. The reduction in EAT volume was greater in patients with a history of persistent AF than those with paroxysmal AF. A larger Japanese study retrospectively assessed 247 patients who underwent cryoballoon ablation for AF, assessing EAT by 128-detector-row computed tomography (CT) at baseline and at 6 months, using a threshold of −200 to −15 Hounsfield units (HU) [65]. Nearly all patients were in sinus rhythm at 6 months; both total and LA-EAT volume decreased significantly (−4.0% and −6.0%, respectively). While reductions were modest, the cohort had a low baseline BMI (23.8 kg/m^2^) and low baseline EAT volume. Another Japanese study prospectively enrolled 120 patients across two centres, imaging EAT by 320-detector-row CT at baseline and one year after radiofrequency ablation for AF, using an EAT threshold of −200 to −50 HU [66]. In patients free of arrhythmia recurrence, total EAT volume decreased by 11.2% and LA-EAT volume by 9.7%. Conversely, total and LA-EAT volume increased by 18.8% and 16.7% among those with recurrence, respectively.

Cumulatively, these studies raise an important possibility that *EAT begets AF begets EAT* [67]. Several mechanisms have been proposed to explain EAT changes after catheter ablation including direct thermal injury, microvascular disruption within the EAT, and ablation-induced inflammation. A plausible explanation is restoration of sinus rhythm with consequent reversal of atrial myopathy, though this has yet to be directly demonstrated. Sinus rhythm has been shown to improve atrial volumes and promote favourable functional remodelling [68]. Atrial natriuretic peptide, secreted by atrial myocytes under mechanical stress, reduces with sinus rhythm restoration [69]. In combination with reduced pro-inflammatory markers such as high-sensitivity C-reactive protein and interleukin-6 [70,71], this shift in the local inflammatory milieu could plausibly alter peri-atrial EAT composition and thus volume, although this mechanism requires further elucidation.

Several limitations from published studies concerning the effect of AF ablation on EAT should be considered. Studies have mostly been retrospective and small-to-modest in sample size. Imaging modality for EAT analysis varied between studies. MRI offers lower spatial resolution and has less standardised quantification compared to CT, although more recent standardised MRI-based approach has shown high reproducibility [72]. In CT-based studies, the Hounsfield unit threshold for EAT delineation varied considerably [65,66]. Additionally, while studies have focused primarily on EAT volume, fat attenuation complements assessment of tissue composition and is increasingly used as a surrogate marker of local adipose inflammation. Quiescent, lipid-rich adipocytes are associated with lower attenuation, while inflammation, lipolysis, and less differentiated adipocytes increase attenuation. Peri-atrial EAT attenuation has been linked to the presence of AF [73]. Also, EAT changes may be confounded by weight loss; the specific contribution of sinus rhythm control to EAT change remains uncertain. Finally, these studies have used thermal energy for ablation and it remains unclear whether these findings extrapolate to pulsed field ablation (PFA) which uniquely achieves cell apoptosis and electrical isolation via irreversible electroporation through the delivery of ultra-short, high-voltage electrical impulses within a localised field [74]. Because direct thermal injury is not the primary mechanism of PFA lesion formation, evaluating EAT change after PFA would effectively exclude this pathway as a potential contributing mechanism.

The interplay between AF burden, underlying atrial myopathy, and EAT composition may help predict response to catheter ablation. Standardised imaging and EAT analysis are still needed. The effect of PFA on EAT remains unknown. Future studies should aim to delineate the direct effects of ablation from the downstream effects of durable sinus rhythm control which may be a determinant of the dynamic relationship between AF and EAT.

## 5. Body Mass Index and AF

Observational studies concerning obesity and AF have influenced guidelines recommending weight reduction in overweight and obese patients. A seminal study from the Framingham cohort investigated 5282 participants without baseline AF and categorised them by BMI [26]. Across a mean follow-up of 13.7 years, there was a 4% (95% CI: 1–7%) increase in incident AF risk per BMI unit after adjusting for cardiovascular risk factors. These findings have been corroborated by later observational studies investigating links between obesity and AF. A meta-analysis examined cohort and case–control studies to quantify the risk of AF according to BMI [75]. Amongst cohort studies, there was a 29% increase (OR: 1.29, 95% CI: 1.23–1.36) in overall risk of AF for every five-unit increase in BMI. As for post-operative AF, a five-unit increase in BMI was associated with a 10% increase in risk of post-operative AF (OR: 1.10, 95% CI: 1.04–1.17). The risk of AF recurrence post-ablation increased by 13% for every five-unit increase in BMI (OR: 1.13, 95% CI: 1.06–1.22). Current observational data suggest that BMI is a key risk factor for AF and a crucial target of risk factor modification strategies in the management of AF.

## 6. Randomised Trials of Weight Management in AF

Given the supportive observational studies showing the association between BMI and AF, it has been reasonably concluded that weight reduction affects incident and recurrent AF. An observational Australian study (LEGACY) offered a weight management strategy to 355 patients with AF and a BMI ≥ 27 kg/m^2^ and followed up with them for a median of ~4 years [9]. Patients received a structured motivational and goal-directed programme with in-person counselling and meal plans and maintained a diet and physical activity diary. For those who had minimal (<3%) weight reduction at 3 months, they were prescribed very-low-calorie meal replacement sachets. The physical activity prescription was 20 min of low-intensity exercise three times per week and increasing to ≥200 min of moderate-intensity activity per week over time. Other risk factors were screened including hypertension, hyperglycaemia, OSA, alcohol excess and tobacco use. In a selected cohort (1415 eligible patients, 355 patients enrolled; 25%), only 38% of patients achieved and maintained the target weight reduction of ≥10%. They reported a substantial difference in freedom from AF and AF symptom severity with a stepwise improvement in outcomes according to the degree of weight reduction (<3%, 3–9% and ≥10%). The same group of investigators also conducted a small, randomised trial of 150 patients on an intention-to-treat basis and reported that a weight management intervention similarly improved AF-related symptoms and AF duration, although hampered by loss of follow-up (while 73% completed a 12-month follow-up, only 54% of participants who completed a 15-month follow-up were included in their primary analysis) [8].

However, weight reduction strategies evaluated in other randomised clinical trials have had limited success in reducing AF or improving quality of life (Table 1). The PRAGUE-25 trial was a multicentre noninferiority trial that randomised 203 patients with either paroxysmal or persistent AF and a BMI of 30–40 kg/m^2^ to either catheter ablation or lifestyle modification with anti-arrhythmic drugs [13]. Patients randomised to the lifestyle modification arm were treated by a team of dietary specialists and physiotherapists for 4 weeks after enrolment, had regular in-person and telephone consultations, and recorded their caloric intake and exercise activity on a mobile phone application. Enrolled patients had morbid obesity (BMI of 35 kg/m^2^), about 40% had persistent AF and about half of patients who received ablation used pulsed field technology. At 1-year follow-up including a blanking period, the criteria of noninferiority was not met; patients who received catheter ablation had markedly higher AF-free survival (73%) compared with those in the lifestyle modification group (34.6%). However, the lifestyle modification arm only achieved an overall mean weight reduction of 5.8% (6.4 kg). While weight changes correlated with improved glycaemia, lipid profile and maximal oxygen consumption (VO2 max), it was far short of the guideline-proposed target of ≥10%. Despite the relatively resource- and time-intensive nature of their lifestyle modification intervention, their study reflects the challenges with achieving clinically significant weight reduction. A subsequent per-protocol analysis reported that patients in the highest tertile of weight reduction (>8.3%) did not differ significantly in AF burden from the catheter ablation group while those in the second or third tertile had significantly higher AF burden than the catheter ablation group. This should be interpreted as a hypothesis-generating finding, as a nonsignificant difference does not confirm equivalence between weight reduction and catheter ablation in reducing AF burden [76].

In the more recent LOSE-AF trial, investigators from the United Kingdom randomised slightly older patients (inclusion criteria: 60–85 years) with a history of persistent AF referred for DC cardioversion and a BMI ≥ 27 kg/m^2^ to a weight reduction strategy or standard care [12]. Their weight reduction intervention was similarly intense with a structured 32-week programme through a commercial provider, individual counselling with behavioural coaching and provision of low-caloric formula meal products aiming for a caloric intake of between 800 and 1200 kcal/day. While their primary outcome was AF symptom severity assessed at 8 months, their initial protocol was revised from a primary endpoint of AF recurrence due to slow recruitment. As such, their proposed sample size was revised from 180 to 118 patients. At 8 months, patients in the weight reduction arm achieved 9.7% weight reduction compared to 3.1% in the control arm. The prespecified clinically relevant between-group difference was four points. The mean AFSS symptom severity score was 7.9 in the weight reduction arm as compared to 8.9 in the control arm, yielding a between-group difference of −0.9 (95% CI: −3.3 to 1.4, *p* = 0.43). Since the confidence interval excludes the prespecified clinically relevant difference, it was concluded that the weight reduction did not result in any clinically meaningful difference in the primary outcome. This finding was also consistent on subgroup analyses according to sex, age and BMI. No between-group differences were noted for physical performance, cardiac structure, blood pressure or serum cardiometabolic markers. The lack of differences in these surrogate markers conventionally associated with weight reduction could signal a type II statistical error. While patients in the weight reduction arm achieved 9.7% weight reduction, those in the control arm also lost >3% weight which could limit between-group differences. It should be noted that in the observational LEGACY study, the Kaplan–Meier curve for AF-free survival without the use of rhythm control strategies showed divergence of curves for the group that achieved ≥ 10% weight reduction, whereas those who achieved <3% or 3–9% weight reduction had very similar outcomes. Furthermore, patients in their study who achieved >10% weight reduction actually had an average overall weight reduction of 15.8% (16 kg). This source of observational data may suggest that in the LOSE-AF trial, a 9.7% weight reduction may still be relatively inadequate to improve AF outcomes. There remains no randomised evidence to establish a biological threshold for percentage weight reduction that achieves clinically meaningful outcomes in patients with AF. It is also possible that older patients (median: 68 years) recruited in their study relative to other clinical trials of AF ablation may be less responsive to intentional weight reduction given the possible competing risk of frailty and sarcopenia [77,78]. Additionally, 44–51% of patients had persistent AF and their pre-existing burden of AF may reflect an underlying advanced atrial myopathy [79]—a substrate likely less responsive to weight reduction intervention.

The SORT-AF study was a multicentre trial in Germany that evaluated the role of weight reduction compared with standard care in patients with BMI of 30–40 kg/m^2^ undergoing thermal AF ablation [14]. All patients were screened for OSA and received an implantable loop recorder. The weight reduction programme involved twice-a-month medical attendance, regular nutrition advice and physical training for 6 months in the context of a specialised multidisciplinary obesity department supervised by an endocrinologist. Enrolment occurred over 6 years but only 142 patients were recruited of which nine patients dropped out. The weight reduction group achieved a modest 3.91% weight reduction (4.6 kg) with non-adherence to intervention reported at 33% at 12 months. In both groups, AF burden was reduced but with no significant between-group differences. In fact, there was a numerically higher rate of repeat ablation in the weight reduction group (20%) compared with standard care (13%).

It is acknowledged that these trials differ in several ways including their comparator arm, lifestyle intervention programme and patient population. Furthermore, it is difficult to isolate the role of weight reduction alone compared with broader, more comprehensive lifestyle modification that are often integrated in the treatment. Nevertheless, these trials collectively underscore how challenging it is to achieve weight reduction with translatable clinical benefits even in an intensely monitored trial context. Despite the labour- and time-intensive nature of these structured weight reduction programmes, most participants did not meet guideline-recommended ≥10% weight reduction target. Our approach to weight reduction in both clinical trial and real-world settings for patients with AF will need to be reconsidered in light of the findings from these recent trials.

## 7. Role of Incretin-Based Therapies

GLP-1 RAs were originally developed for treatment of T2DM but have since been shown to markedly reduce weight alongside other cardiometabolic benefits. Metabolic effects are achieved by mimicking GLP-1, an incretin. GLP-1 RAs increase insulin secretion in a glucose-dependent manner [80,81], slow gastric emptying [80,82,83], reduce appetite and promote satiety [84,85]. The effects of GLP-1 RAs such as semaglutide, liraglutide, exenatide and efpeglenatide induce weight reduction of about −7.1 kg (95% CI: −9.2 to −5.0 kg) with treatment durations ranging from 14 weeks to 3 years [86]. They have led to sustained weight reduction while on treatment [87]. Although bariatric surgery confers a higher degree of weight reduction compared to GLP-1 RAs [88], it is invasive and generally reserved for patients with morbid obesity when less invasive options fail, making GLP-1 RAs an attractive upfront alternative [89].

Incretin-based therapies collectively include pure GLP-1 RAs, dual receptor agonists and triple receptor agonists which have GLP-1, glucose-dependent insulinotropic polypeptide (GIP) and glucagon effects. Pure GLP-1 receptor agonists include liraglutide, dulaglutide, semaglutide, exenatide and lixisenatide [90]. Tirzepatide has a dual GIP/GLP-1 effect and has been approved by the FDA and EMA for T2DM and weight management for obesity and by FDA for reduction in MACEs in high-risk adults with T2DM [91,92,93]. Survodutide is an investigational dual GLP-1/glucagon receptor agonist currently in Phase 3 trials. Retatrutide is an investigational triple receptor (GLP-1, GIP and glucagon) agonist currently in Phase 3 trials.

GLP-1 RAs and other incretin-based therapies improve cardiovascular risk factors and outcomes in patients with obesity. GLP-1 RAs improve glycaemic control in patients with T2DM and lower HbA1c [94]. They reduce blood pressure and have a favourable effect on lipid profile, reducing triglyceride, ApoB, total cholesterol and very low-density lipoprotein levels [95]. Semaglutide has also been shown to reduce HFpEF symptom burden in patients with AF [96]. GLP-1 RAs significantly reduce EAT thickness [95,97]. Multiple trials of GLP-1 RAs and other incretin-based therapies have been shown to reduce MACEs. Trials utilising liraglutide, semaglutide and dulaglutide have demonstrated superiority over placebo in reducing the risk of MACEs in patients with T2DM [98,99,100], with a trial involving semaglutide demonstrating efficacy in reducing MACEs even in patients without T2DM [101]. FDA approval has been granted in adults with T2DM for MACE reduction for semaglutide [102,103], dulaglutide [104], liraglutide [105] and tirzepatide [91]. Additionally, semaglutide has been FDA-approved in overweight or obese adults with CVD for MACE reduction independent of T2DM [106].

The role of incretin-based therapies in managing AF in adults with obesity is less clear. While some prospective cohort studies have investigated incretin-based therapies for AF in patients with obesity, no RCTs have been conducted evaluating AF as the primary endpoint. A propensity-matched, retrospective cohort study utilising the TriNetX database found that GLP-1 RAs reduced the primary composite of cardioversion, new anti-arrhythmic drug therapy and the need for repeat AF ablation (HR: 0.72, 95% CI: 0.65–0.80, *n* = 3116) in patients with AF and a BMI > 30 kg/m^2^ undergoing catheter ablation [107]. The duration of treatment with GLP-1 RAs was not delineated, but was defined as GLP-1 RAs at the time of catheter ablation. Another propensity-matched retrospective cohort study also using the same database reported that GLP-1 RAs within 1 year before or up to 6 months after AF ablation were associated with lower AF recurrence and lower all-cause mortality post-ablation (HR: 0.82, 95% CI: 0.76–0.88, *n* = 6700) [108]. A single-centre propensity-matched observational analysis derived from a prospective Italian registry found that semaglutide was associated with 48% lower recurrence of AF post-ablation over 18 months of follow-up (HR: 0.52, 95% CI: 0.34–0.78, *n* = 362) [15]. All patients underwent continuous rhythm monitoring using an implantable cardiac monitor post-ablation. Another prospective cohort study in a Chinese population also found that semaglutide reduced the risk of AF recurrence post-ablation by 37% (HR: 0.63, 95% CI: 0.45–0.91, *p* = 0.019) [109]. Importantly, despite propensity-matching, there may be unmeasured confounding variables that affect the conclusions of these studies, such as weight-loss motivation and engagement with care that are difficult to account for, limiting the generalisability and definitive conclusions of these findings. Randomised trials evaluating GLP-1 RAs for AF are limited. The LEAF trial examined liraglutide in patients with AF undergoing catheter ablation [110]. The primary endpoint was change in LA-EAT volume; secondary outcomes included total EAT volume and recurrent AF at 1 year. They found that liraglutide was not associated with a significant change in LA-EAT or EAT, although it reduced AF recurrence during 12 months of follow-up (HR: 0.33, 95% CI: 0.14–0.77, *n* = 59) [110]. This finding remains hypothesis-generating as the study was not powered for AF recurrence and deserves further investigation as to whether this effect is mediated by LA-EAT or by other mechanisms.

In summary, the effects of incretin-based therapies on AF are still uncertain. Observational studies suggest that incretin-based therapies may be a beneficial adjunct to ablation to reduce AF recurrence independent of weight reduction, but evidence remains limited given possible confounding. Adequately powered randomised trials evaluating AF as a primary endpoint are needed to examine their potential adjunctive role in the management of AF. Future studies such as TIRO-AF (NCT06802081, expected enrolment of 100 participants), SEMINAL-AF (NCT06499857, expected enrolment of 200 participants) and SPICE-AF (NCT07389941, expected enrolment of 240 participants) will be informative on this topical issue.

## 8. Lifestyle Risk Factor Modification Clinics for AF

Weight management is only one of many lifestyle risk factors that contribute to AF. Regular physical exercise, alcohol reduction and abstinence, blood pressure control and OSA are modifiable contributors to AF [111,112,113,114]. When addressed together, these factors are likely to cumulatively affect AF-related clinical outcomes. This underscores the need to better define the role of comprehensive LRFM clinics, which target upstream contributors alongside patient education and medication adherence [115]. Most of the existing evidence comes from nurse-led models, at times supported by a multidisciplinary team delivering individualised lifestyle interventions within a patient-centred framework. More recently, several RCTs have examined this approach specifically in patients undergoing AF ablation (Table 2) [16,116]. Beyond their direct clinical benefit, multidisciplinary clinics also reduce the burden placed on electrophysiologists to manage lifestyle risk factors within the constraints of routine outpatient consultations.

The first major trial to evaluate the role of integrated nurse-led chronic care in patients with AF was conducted in the Netherlands that randomised 712 outpatients with AF to nurse-led care versus usual cardiologist-only care [117]. The nurse-led care model was derived from a chronic care model that utilised nurse specialist education in combination with a guideline-informed decision-support software. During a mean follow-up of 22 months, nurse-led care reduced the composite primary endpoint of cardiovascular hospitalisation and death (14.3% vs. 20.8%, HR: 0.65, 95% CI: 0.45–0.93, *p* = 0.017). The trial appeared appropriately powered, although it still had a broad 95% CI, was limited to a single centre and adopted a specific software to guide decision-making that may not be widely available elsewhere.

The findings from the abovementioned single-centre trial [117] were not replicated by the RACE4 investigators [118]. In this multicentre trial also conducted in the Netherlands, 1375 patients were randomised to nurse-led care using the same dedicated software versus usual cardiologist care and followed up with for a median of 37 months. Despite better guideline adherence in the nurse-led arm (61% vs. 26% cumulative adherence), the trial showed no significant difference for the primary composite endpoint of cardiovascular death and hospitalisation (9.7%/year vs. 11.6%/year, HR: 0.85, *p* = 0.12). Importantly, the events were adjudicated by an independent committee unaware of the randomised treatment assignments. However, there was significant heterogeneity according to centre experience: nurse-led care was beneficial in experienced centres (HR: 0.52) but not in less-experienced ones (HR: 1.24, interaction *p* < 0.001). Such findings highlight the realities of implementation science—what can be achieved in a trial context with rigorous follow-up and intensive consultation may not be replicable in less-experienced centres, let alone in a real-world setting limited by time and resource constraints. Additionally, across the eight hospitals involved, their total number of patients recruited over the enrolment period would be approximately 34 patients per year per hospital; this is far shy of the approximately 356 patients per year recruited in the initial single-centre trial [117] which was likely far more experienced both in recruitment and in delivery of nurse-led care.

A Belgian trial evaluated nurse-led care which utilised advanced practitioner nurse consultations after AF ablation [116]. In this small (65 patients) single-centre study, their intervention, which consisted of an educational session, three consultations over 6 months and telephone accessibility coordinated by the nurse practitioner, resulted in a significantly lower AF recurrence at 6 months (13.5% vs. 39.4%, *p* = 0.014), higher AF knowledge and patient satisfaction. Reduced alcohol intake and higher physical activity was also observed in the intervention group. A notable limitation of this trial concerns their primary endpoint—the analysis of freedom from AF did not account for a blanking period post-ablation and the separation in the Kaplan–Meier curve occurs only in the first two months with a plateau in both groups thereafter. The implementation of a typical blanking period (historically 3 months) might have reduced or removed the difference in their primary endpoint, but this cannot be confirmed without a formal reanalysis. It is also questionable whether educational counselling regarding typical lifestyle risk factors would confer a significant benefit in the acute period.

Another trial conducted in the Netherlands (POP-AF) evaluated the role of an intensive, multidisciplinary, nurse-led integrated lifestyle programme delivered before PFA for AF [16]. In this single-centre trial, 145 patients with at least one modifiable risk factor (obesity, hypertension, dyslipidaemia, diabetes, smoking, excess alcohol, or OSA) were randomised to an integrated lifestyle treatment or standard care. Notably, the lifestyle treatment arm was comprehensive. The nurse-led clinic involved monitoring of cardiometabolic parameters, OSA screening, dedicated software for decision-making, and structured interviews for lifestyle behaviours including physical inactivity, alcohol consumption, smoking, hypertension, hyperlipidaemia, hyperglycaemia and OSA. Patients also received a supervised weight management programme and structured AF education involving a multidisciplinary team involving cardiologists, respiratory physicians, sleep medicine physicians, endocrinologists, general practitioners, dieticians, physiotherapists, psychologists and smoking-cessation counsellors. Overall, the integrated lifestyle treatment group had about a 50% relative risk reduction for the composite of repeat ablation and cardioversion from enrolment up to 12 months post-ablation (240 vs. 492 events/1000 patient–years, RR: 0.49, 95% CI: 0.30–0.78, *p* = 0.004), with reduction in both repeat ablation (RR: 0.43, *p* = 0.045) and cardioversion (RR: 0.52, *p* = 0.031) individually. It is not clear whether the electrophysiologists involved in the decision to refer for repeat ablation or cardioversion or the decision to stop anti-arrhythmic drugs were blinded to the patients’ initial assigned treatment arm. Nevertheless, it is remarkable that such a comprehensive multidisciplinary clinic could be made available to patients with AF and it would be important to see real-world data regarding this model of care, particularly in more resource-strained settings.

A multicentre Australian trial provided the randomised confirmation of the earlier ARREST-AF cohort study [10], comparing a comprehensive LRFM clinic against usual care in 122 patients with elevated BMI and cardiometabolic risk factors undergoing first-time ablation, with continuous implantable loop recorder monitoring over 12 months [17]. The LRFM group, managed in a dedicated physician-led clinic targeting blood pressure, weight (structured meal plans and exercise), lipids, glycaemia, OSA, smoking, and alcohol, achieved greater freedom from atrial arrhythmia (61.3% vs. 40.0%, HR: 0.53, 95% CI: 0.32–0.89, *p* = 0.03) and lower AF symptom burden, alongside substantial improvements in body weight (−9.0 kg), waist circumference, and systolic blood pressure relative to usual care.

A challenge in comparing these trials is the heterogeneity in the delivery of LRFM clinics themselves. Most share a common theme including a structured patient interview and educational component covering lifestyle risk factors, AF pathophysiology and the importance of medication adherence, often supplemented by cardiovascular screening for lipid abnormalities, glycaemic control, blood pressure, and OSA. While the nurse-led clinic was the predominant delivery model, some trials involved a broader multidisciplinary team spanning cardiology, respiratory, sleep medicine, endocrinology, primary care, dietetics, physiotherapy, psychology, and smoking-cessation counselling. Contact intensity also varied widely, from telephone check-ins to face-to-face reviews scheduled roughly every three months, with the overall LRFM intervention sometimes running for as long as six months before ablation was even performed [16]. Some trials also use dedicated decision-support software that tailors AF and cardiovascular management to the individual’s symptom profile, AF subtype, and stroke risk. Thus the implementation of LRFM clinics in any individual health service will need to consider practicalities of staff, duration, intensity and cost. Additionally, it should be acknowledged that many of the positive trials have arisen from single-centre experiences or from a single Australian group, and that participants across these trials likely represent a self-selected population more motivated and receptive to lifestyle change and may not be representative of the broader population with AF.

## 9. Future Direction

Moving forward, the role of obesity in AF will need to be considered within a holistic framework recognising the contribution of CKM domains to the disease substrate. This is supported by numerous trials showing benefits of addressing AF from a comprehensive LRFM clinic in a multidisciplinary setting. This lens changes obesity from a single metric of BMI to a multidimensional phenotype including patient demographics, adiposity measured through various visceral and subcutaneous adiposity metrics, cardiometabolic markers such as lipid profile, glycaemia, blood pressure and OSA, cardiovascular imaging parameters such as LA volume and EAT characteristics, and electroanatomic mapping data such as LA endocardial conduction velocity and voltage. While the number of parameters to be considered reflects the complexity of AF, it is possible to better understand their individual and collective role in contributing to AF substrate particularly in the era of artificial intelligence.

**Figure 2:**
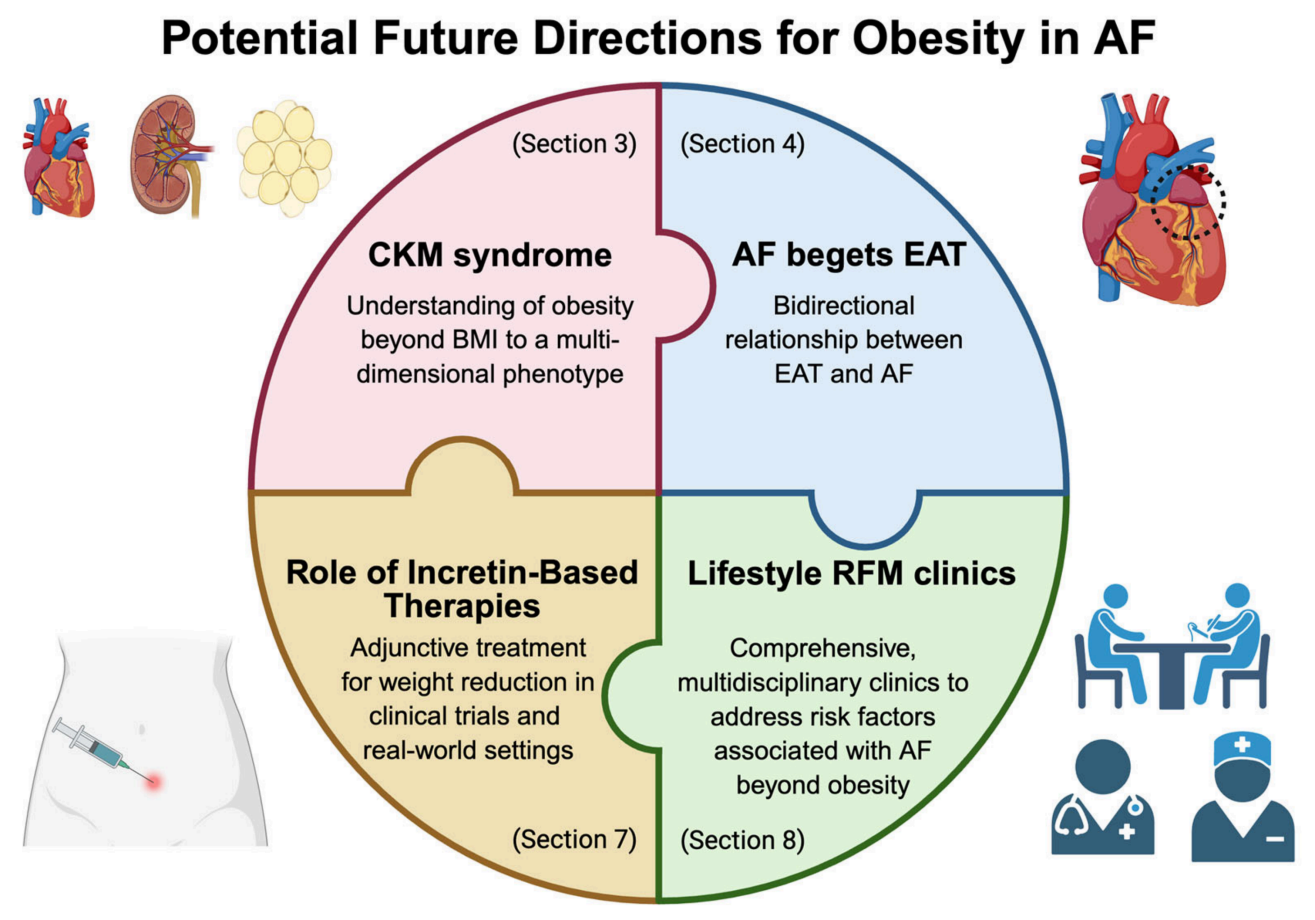
Potential future directions for managing obesity in AF. AF, atrial fibrillation. CKM, Cardiovascular–kidney–metabolic (syndrome). GLP-1 RA, Glucagon-like peptide-1 receptor agonist. RFM, Risk factor modification. Created using Biorender.com with licenced permission.

There are several further research priorities that would be most clinically impactful in our understanding of obesity and AF (Figure 2). Firstly, we need an improved understanding of the mechanistic and biological role of how *EAT begets AF begets EAT*, that is, the perpetuating role that AF has in affecting EAT and conversely, the role that sinus rhythm restoration has in reversing the pro-inflammatory and pro-fibrotic phenotype that EAT can pathologically shift towards. Second, if we are to isolate the role of weight reduction in AF, our current approach to weight reduction in clinical trials will need to change. They will very likely incorporate various incretin-based therapies alongside lifestyle changes to induce clinically meaningful and sustained weight reduction. Upcoming aforementioned trials investigating incretin-based therapies to potentially reduce risk of AF will be insightful [119,120,121]. Further clarity regarding the role of the obesity paradox, particularly in older patients with a tendency towards an atrial myopathy, is needed to determine both the safety and efficacy of weight reduction strategies. Third, more real-world data and experience are needed regarding a comprehensive model of care that addresses lifestyle risk factors, AF education and medication adherence implemented in various healthcare settings, while also accounting for the local patient demographics, health economics and cultural influences.

## 10. Conclusions

The landscape of weight management in patients with AF is evolving. The role of EAT is still being elucidated, although a bidirectional relationship with AF is increasingly plausible. While several RCTs focused on weight management alone have failed to show clinically meaningful benefits in relation to AF outcomes, future trials will define how incretin-based therapies can be integrated into AF management. The emphasis in both clinical trial and real-world settings will need to shift towards the incorporation of comprehensive LRFM clinics that understand AF beyond an isolated arrhythmia but as a marker of overall cardiometabolic health.

## Figures and Tables

**Table 1:** Recent randomised clinical trials focused on weight reduction for atrial fibrillation. NB1: While these trials are not directly comparable due to differing comparator arms and endpoints, this table provides a useful summary of the trials mentioned in the text. NB2: The original intervention and control arms for PRAGUE-25 were catheter ablation and LFM + AAD respectively. To make formatting consistent and to improve readability, they have been reversed in this table.

	SORT-AF (2021) [14]	PRAGUE-25 (2025) [13]	LOSE-AF (2026) [12]
**Study design**	Weight reduction strategy vs. usual care in patients undergoing AF ablation	Weight reduction strategy and AADs vs. catheter ablation	Weight reduction strategy vs. usual care in patients with AF who underwent electrical cardioversion
**Intervention type**	Structured weight reduction programme (nutrition + physical training) in addition to usual care	Directed weight loss + physical exercise + alcohol reduction (collectively LFM + AAD)	Low-calorie diet (800–1200 kcal/d) + behavioural support
**Control arm**	Usual care	Catheter ablation	Usual care
**Primary outcome**	AF burden between 3 and 12 months after first AF ablation measured by implantable loop recorder	AF freedom between 3 and 12 months after catheter ablation or initiation of weight reduction programme measured on Holter monitor or ECG	Symptom severity subscale of Atrial Fibrillation Severity Scale (AFSS)
**Duration of treatment**	6 months	Ongoing throughout follow-up (≥12 months)	32 weeks (~8 months)
**Duration of follow-up**	12 months	≥12 months	8 months
**Number of patients**	133	203	118
**Age, years**	58.7/62.1	60/60	68.5/68.3
**Females, %**	35.8/37.9	31.1/32.0	33.9/32.2
**Persistent AF, %**	67.2/48.5	39.8/39.0	100.0/100.0
**Longstanding persistent AF, %**	Not reported	Not reported	44.1/50.8 (longstanding persistent)
**Mean weight reduction in weight reduction group**	3.91%	5.8%	9.7%
**Result**	OR 1.143, 95% CI 0.369–3.613, *p* = 0.815	% AF freedom: 34.6% in LFM + AAD group, 73.0% in CA group p_noninferiority_ of LFM + AAD = 0.99, p_superiority_ of LFM + AAD < 0.001	Mean difference in AFSS symptom severity score = −0.9, 95% CI −3.3 to 1.4, *p* = 0.43

Data shown as weight reduction group/comparison group where appropriate as stated. This applies to the rows “Age, years”, “Females, %”, “Persistent AF, %” and “Longstanding persistent AF, %”. AF, atrial fibrillation; AAD, anti-arrhythmic drug; CA, catheter ablation; LFM, lifestyle modification.

**Table 2:** Randomised clinical trials of lifestyle risk factor modification clinics.

	Hendriks et al. [117]	RACE 4 [118]	Vanharen et al. [116]	ARREST-AF [17]	POP-AF [16]
**Year Published**	2012	2020	2023	2025	2026
**Study design**	Nurse-led care vs. usual care in patients with AF	Nurse-led care vs. usual care in patients with AF	Advanced practitioner nurse-led care vs. usual care in patients undergoing AF ablation	Lifestyle and risk factor modification vs. usual care in patients undergoing first-time AF ablation	Nurse-led integrated lifestyle clinic vs. standard pre-ablation counselling in patients undergoing first-time AF ablation
**Intervention type**	Nurse-led with CardioConsult AF	Nurse-led with CardioConsult AF	Advanced practitioner nurse-led education, consultations and telephone calls	Physician-led risk factor modification clinic following AHA guidelines	Nurse-led clinic, focusing on a home sleep apnoea test, weight reduction, alcohol reduction, smoking cessation, optimal hypertension and hypercholesterolaemia treatment
**Control arm**	Usual care by cardiologist	Routine outpatient management by cardiologist	Usual care, including offer to participate in cardiac rehabilitation and follow-up by cardiologist	Usual care; patients given information on risk management and self-directed weight management	Standard pre-ablation counselling by treating electrophysiologist
**Primary endpoint**	Composite of cardiovascular hospitalisation and cardiovascular death	Composite of cardiovascular hospitalisation and cardiovascular death	AF recurrence rate at 6-month follow-up	Proportion of patients free from AF during follow-up	Composite of hospitalisations for repeat ablation and DCCV
**Duration of treatment**	Ongoing throughout follow-up	Ongoing throughout follow-up	6 months	12 months	5 months
**Duration of follow-up**	22 months	37 months	6 months	12 months	12 months post-ablation
**Number of patients**	712	1375	65	122	145
**Persistent AF, %**	19.1/12.4	Non-paroxysmal AF: 24.7/20.5	31.3/39.4	61.3/56.7	57.3/60.0
**Females, %**	44.7/37.9	32.9/35.4	43.8/45.5	*30.6/35.0*	*33.3/17.1*
**Results**	HR: 0.65, 95% CI: 0.45–0.93, *p* = 0.017	HR: 0.85, 95% CI: 0.69–1.04, *p* = 0.12	OR: 0.277, 95% CI: 0.090–0.851, *p* = 0.025	HR: 0.53, 95% CI: 0.32–0.89	RR: 0.49, 95% CI: 0.30–0.78, *p* = 0.004

Data shown as lifestyle risk factor modification group/control group where appropriate. AF, atrial fibrillation; AHA, American Heart Association; DCCV, direct current cardioversion; HR, hazard ratio; OR, odds ratio.

## Data Availability

No new data were created or analyzed in this study. Data sharing is not applicable to this article.

## Abbreviations

The following abbreviations are used in this manuscript:

AAD	anti-arrhythmic drug
AF	atrial fibrillation
AFSS	Atrial Fibrillation Severity Scale
BMI	body mass index
CI	confidence interval
CKD	chronic kidney disease
CKM	cardiovascular–kidney–metabolic (syndrome)
CPAP	continuous positive airway pressure
CT	computed tomography
CVD	cardiovascular disease
DCCV	direct current cardioversion
EAT	epicardial adipose tissue
ESRF	end-stage renal failure
GIP	glucose-dependent insulinotropic polypeptide
GLP-1 RA	glucagon-like peptide-1 receptor agonist
HR	hazard ratio
HU	Hounsfield unit
LA	left atrial/atrium
LFM	lifestyle modification
LRFM	lifestyle risk factor modification
LV	left ventricular
MACE	major adverse cardiovascular event
MRI	magnetic resonance imaging
OR	odds ratio
T2DM	type 2 diabetes mellitus
